# Stable Schizophrenia Patients Learn Equally Well as Age-Matched Controls and Better than Elderly Controls in Two Sensorimotor Rotary Pursuit Tasks

**DOI:** 10.3389/fpsyt.2014.00165

**Published:** 2014-11-24

**Authors:** Livia J. De Picker, Claudia Cornelis, Wouter Hulstijn, Glenn Dumont, Erik Fransen, Maarten Timmers, Luc Janssens, Manuel Morrens, Bernard G. C. Sabbe

**Affiliations:** ^1^Collaborative Antwerp Psychiatric Research Initiative (CAPRI), University of Antwerp, Antwerp, Belgium; ^2^University Psychiatric Hospital St. Norbertushuis, Duffel, Belgium; ^3^Donders Institute for Brain, Cognition and Behaviour, Radboud University Nijmegen, Nijmegen, The Netherlands; ^4^StatUA, University of Antwerp, Antwerp, Belgium; ^5^Janssen Research and Development, Janssen Pharmaceutica N.V., Beerse, Belgium

**Keywords:** rotor pursuit, schizophrenia, motor skills, learning curve, aging and cognitive function, procedural learning, motor learning

## Abstract

**Objective:** To compare sensorimotor performance and learning in stable schizophrenia patients, healthy age- and sex-matched controls and elderly controls on two variations of the rotary pursuit: circle pursuit (true motor learning) and figure pursuit (motor and sequence learning).

**Method:** In the circle pursuit, a target circle, rotating with increasing speed along a predictable circular path on the computer screen, must be followed by a cursor controlled by a pen on a writing tablet. In the eight-trial figure pursuit, subjects learn to draw a complex figure by pursuing the target circle that moves along an invisible trajectory between and around several goals. Tasks were administered thrice (day 1, day 2, day 7) to 30 patients with stable schizophrenia (S), 30 healthy age- and sex-matched controls (C), and 30 elderly participants (>65 years; E) and recorded with a digitizing tablet and pressure-sensitive pen. The outcome measure accuracy (% of time that cursor is within the target) was used to assess performance.

**Results:** We observed significant group differences in accuracy, both in circle and figure pursuit tasks (E < S < C, *p* < 0.01). Strong learning effects were found in each group. Learning curves were similar in circle pursuit but differed between groups in figure pursuit. When corrected for group differences in starting level, the learning gains over the three sessions of schizophrenia patients and age-matched controls were equal and both were larger than those of the elderly controls.

**Conclusion:** Despite the reduced sensorimotor performance that was found in the schizophrenia patients, their sensorimotor learning seems to be preserved. The relevance of this finding for the evaluation of procedural learning in schizophrenia is discussed. The better performance and learning rate of the patients compared to the elderly controls was unexpected and deserves further study.

## Introduction

The functional outcome of schizophrenia patients is highly impacted by the severity of their cognitive symptoms and their capacity to learn new skills (1). Two variants of learning are generally distinguished: declarative and procedural learning, the latter referring to skill, habit, or knowledge acquisition that occurs in an implicit manner, i.e., automatically and outside of conscious awareness (2). Sensorimotor learning, the incremental spatial and temporal accuracy of movements with repetition, represents a form of procedural learning involving different corticostriatal circuits from those in other forms, such as probabilistic classification (3).

Designed as a tool to evaluate motor learning, the rotor pursuit task has been first used in 1947 (4). It measures the ability to keep a stylus on a rotating target, requiring motor control over the proximal upper limb (including shoulder–elbow control and postural control), as well as the ability to continuously process and adapt to sensory (visual and proprioceptive) feedback. Rotor pursuit performance is known to be altered in several pathologies involving the basal ganglia; impaired performance has been demonstrated in Huntington’s and Parkinson’s disease and enhanced performance in the early trials of the task is seen in patients with obsessive–compulsive disorder (5). The key substrate of the basal ganglia’s involvement in sensorimotor performance and learning is represented by their extensive reciprocal connections to motor and premotor areas of the frontal lobe, implicated in planning and execution of movements (6). Besides the role of the striatal–cortical circuitry, which is considered particularly important in learning operated through the implicit mode, tracts involving the (pre)motor cortex, the supplementary motor area, and the cerebellum are also implicated in the generation of precise forces and spatial knowledge required for learning new motor skills (7).

In contrast to declarative tasks, in which schizophrenia patients have consistently shown impaired performance and learning compared to healthy controls, procedural learning has been less well studied. Both corticofrontal and striatal involvement are presumed in the pathophysiology of schizophrenia, and abnormal dopamine regulation within the basal ganglia is thought to contribute to the psychotic symptoms of the disease. However, studies examining patients with schizophrenia on the pursuit rotor motor-skill learning task have so far produced mixed results when comparing both general performance and learning rate of patients to healthy controls (see Table 1) (8–15). Reasons for the conflicting results may reflect methodological differences including in instrumentation, in equating for initial performance, in number of trials administered, or influences of intrinsic moderating variables, such as general intellectual capacity and declarative memory, as well as the effect of psychotropic drugs. It is also debated whether any impaired performance on the rotor pursuit may be related more to underlying psychomotor deficits or to general cognitive decline, both features of schizophrenia (16).

**Table 1:** **Summary of sensorimotor skill studies with Pursuit rotor task in schizophrenia patients**.

Author (year)	Huston and Shakow (1949)^(8)^	Goldberg et al. (1993)^(9)^	Granholm et al. (1993)^(10)^	Clare et al. (1993)^(11)^	Schwartz et al. (1996)^(12)^	Kern et al. (1997)^(13)^	Weickert et al. (2002)^(14)^	Gomar et al. (2011)^(15)^
Version	Contact	Contact	Photoelectric	Not specified	Contact	Photoelectric	Not specified	Digital

Design	2 blocks × 5 trials × 10 s	3 blocks × 5 trials × 20 s	6 blocks × 4 trials × 20 s	5 blocks × 6 trials × 20 s	6 blocks × 4 trials × 20 s	6 blocks × 4 trials × 20 s	6 blocks	6 blocks × 4 trials × 20 s

Days	d1	d1	d1	d1–d8	d1	d1	d1	d1–d8

*N*	SZ 122, C 60	24 discordant and 7 normal MZ twin pairs	SZ 11, C 11	SZ 11, C 12	SZ 40, C 40 (each 20 elderly, 20 young)	SZ 18, C 15	SZ 35, C 35	SZ 43, C 22

SZ age; mean (range)		31 (17–44)	38.4	42.7 (21–70)	YSZ 33.1 (26–40), ESZ 63.2 (55–70)	36.7	Not specified	46.9 (24–64)

SZ sex M:F		14:10	11:0	7:5	38:2	18:0	Not specified	34:9

RPM	60	30 and 60	45	30	ESZ 40.50, EC 48.75, YSZ 47.25, YC 56.25	SZ 37.2, C 62.7	Not specified	Not specified

Trial 1 matched?	No	No	No	No	Yes	Yes	Not specified	Yes

IQ matched?	No	No	No	No	No	No	Yes (subsample *n* = 14)	Yes (subsample *n* = 22)

Absolute performance difference	SZ < C	SZ = C	SZ = C	SZ < C	SZ < C	SZ = C	SZ < C; IQ matched SZ = C	SZ < C; IQ matched SZ = C

Learning rate difference	Not specified	Not specified	SZ = C	SZ = C	SZ < C	SZ = C	SZ = C	SZ = C

*SZ, schizophrenia patients; C, controls; ESZ, elderly schizophrenia patients; EC, elderly controls; YSZ, young schizophrenia patients; YC, young controls; RPM, rotations per minute*.

Considering the outcomes of previous studies using the rotor pursuit task in schizophrenia, we hypothesized that true sensorimotor learning would be preserved in schizophrenia patients (10, 11, 13–15). However, many tasks that measure procedural learning also include a cognitive aspect, e.g., in the form of an implicit sequence to be learned. It has been postulated that motor and cognitive aspects of procedural tasks are governed by different brain processes; motor or skill learning aspects have been associated with a corticostriatal motor circuit involving the putamen, whereas aspects of cognitive or habit learning are suggested to operate the dorsolateral prefrontal cortex circuit involving the caudate (14). Previous studies that have tried to compare performance on these two aspects have been using combinations of methodologically distinct tasks (e.g., rotor pursuit and a probabilistic classification task, such as the weather prediction task), complicating the direct comparison of their relative outcomes (14).

In this study, we aim to assess the cognitive and motor aspects involved in sensorimotor skill learning in the same pursuit task set up, by using two separate task variations, one of which incorporates also a sequence component.

Furthermore, a longitudinal set up with repeated sessions over several days offers the added value of distinguishing between early (encoding and acquisition) and late (retention/consolidation) phases of sensorimotor learning, as distinguished in literature (7).

An age-related decline in sensorimotor performance and learning on the rotor pursuit has been described (17). Besides the schizophrenia patients and age-matched controls, we, therefore, also included a group of elderly healthy participants to investigate whether the sensorimotor deficits in schizophrenia patients are comparable to those associated with advanced age. We expected both schizophrenia subjects and elderly participants to perform poorer than young control subjects in the sensorimotor rotary pursuit tasks.

## Materials and Methods

### Study design

For all subjects enrolled, the study consisted of an eligibility screening examination (up to 21 days prior) and three cognitive assessment days. The screening examination included baseline assessments of executive functioning (Wisconsin Card Sorting Test; WCST), premorbid IQ (Dutch Adult Reading Test/*Nederlandse Leestest voor Volwassenen*; NLV), and psychomotor speed (measured with a line-copying task on a digitizing tablet; LCT).

Cognitive assessments were made in two subsequent sessions (days 1 and 2), which were separated by overnight sleep. An additional third session was performed on day 7. The pursuit task was part of a cognitive test battery of approximately 90 min that was administered to all subjects in the same way and will be reported elsewhere. The time of day for completion of the cognitive test batteries was comparable on all test days for each subject, but not identical for all subjects.

The study was conducted in accordance with the ethical principles that have their origin in the Declaration of Helsinki and that are consistent with Good Clinical Practices, applicable regulatory requirements, and in compliance with the study protocol. The study protocol was reviewed and approved by the Institutional Ethics Committee.

### Participants

After giving written informed consent, subjects were screened to ascertain their eligibility for the study according to the in- and exclusion criteria specific for the population enrolled. The patient sample consisted of 30 outpatients aged 18–55 with a known history of schizophrenia or schizo-affective disorder (based on DSM-IV criteria) of at least 12 months, as confirmed by the referring psychiatrist. Exclusion criteria were current use of drugs with anticholinergic properties (including tricyclic antidepressants) and benzodiazepines, or comorbid DSM-IV diagnosis of substance dependence within 3 months prior to screening evaluation (except for caffeine and nicotine dependence); patients with a positive drug screen at screening could be included provided they did not meet DSM-IV diagnosis of substance dependence and consented to abstain from illegal drugs at any time during the study. An alcohol breath test and urine drug screening were performed at each of the cognitive assay days. All patients were stably treated with antipsychotic medication for at least 6 weeks, with no more than two different antipsychotic drugs used concurrently. Patients were judged to be in stable clinical condition at the time of testing through subject interview and medical history review by a trained clinician. Symptom severity of patients was rated at screening by a trained psychology assistant using the scale for the assessment of negative symptoms and positive symptoms (SANS-SAPS) (18, 19).

Thirty age- and gender-matched control participants, as well as 30 gender-matched elderly participants (>65 years of age) were recruited from the local community. They met the same exclusion criteria as the patients. They were also interviewed by a clinician to verify that they had no personal history of psychiatric disorders nor first-degree relatives with psychotic disorders and that they were not using any psychotropic medication.

### Pursuit task set up

Based on the classical rotary pursuit task (20), our pursuit rotor (PR) continuous sensorimotor tasks required subjects to follow the movements of a target circle (12 mm in diameter) on the computer screen with a cursor they could control by manipulating a pressure-sensitive pen on a digitizing writing tablet (WACOM1218RE), recording at 200 Hz frequency and 0.2 mm spatial accuracy.

**Figure 1:**
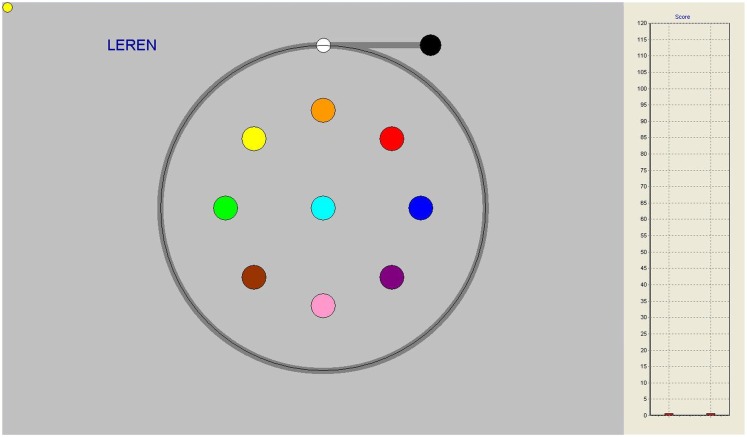
**Circle pursuit task on-screen, gray line indicating pursuit trajectory, not seen by participants**. On the right-hand side, performance bars appeared as feedback after each trial.

In the circle pursuit (CPR) task, the target circle rotates along a predictable circular path with a radius of 7.5 cm (see Figure 1). This task consisted of two trials of 30 s duration with six rotations each. The speed of the target was gradually increased from 10 s per 360° rotation (6 RPM) to 3 s per full rotation (20 RPM).

**Figure 2:**
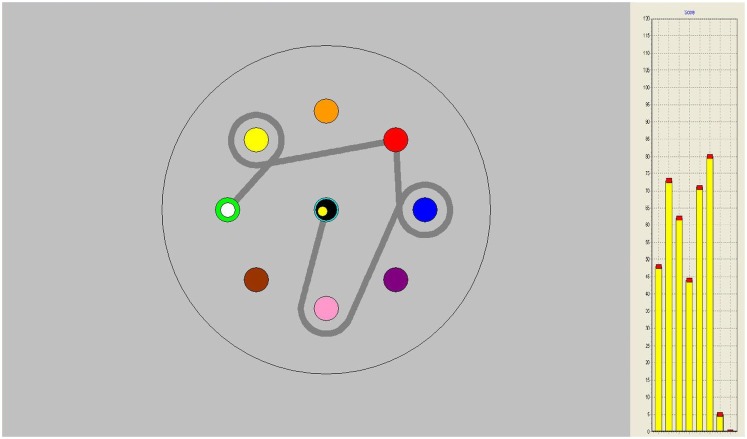
**Figure pursuit task on-screen, gray line indicating pursuit trajectory, not seen by participants**. On the right-hand side, performance bars appeared as feedback after each trial.

The CPR was directly followed by the figure pursuit (FPR) task in which subjects had to follow a trajectory between and around several on-screen goals (see Figure 2). This task can be perceived as learning to draw a complex figure in a so-called “pursuit” condition in which a person is asked to keep the pen cursor on a target circle that moves along the (invisible) trajectory that has to be learned. The start and end positions of the sequence are marked by white and black circles, signaling with a high and low beep, respectively, when the cursor reaches them. This task consisted of eight identical trials of 10 s duration.

Both during circle and figure pursuit, subjects were able to follow their level of performance throughout the task, with vertical score bars appearing on the right side of the screen after each trial, indicating their relative level of target contact (see Figures 1 and 2).

The dependent variable in both task variations was accuracy (% of time that the cursor is within the target circle, higher numbers indicating better performance). The total time of the PR tasks was approximately 3 min.

### Statistical analysis

We performed all statistical analyses in IBM SPSS Statistics for Windows, Version 22.0 (Armonk, NY, USA). Demographic features and baseline assessment results were analyzed using independent samples *T*-tests to evaluate significant group differences. There were some missing data for the Y group on the LCT (*n* = 3) and for the E group on the WCST (*n* = 2) and the LNS (*n* = 1). WCST outcome was defined as the number of categories completed. The movement time (MT) on the LCT was chosen as the relevant outcome measure for psychomotor speed.

The PR performance was quantified by the variable accuracy, and measured in three groups (schizophrenia patients = S, young controls = C, elderly participants = E). We tested each individual repeatedly in three sessions (day 1, day 2, day 7). Within each session, several identical trials were performed (two trials in CPR, eight trials in FPR). There were no missing data on the PR tasks. To provide a measure for the amount of learning over sessions, we computed two learning measures for each session: the mean and the cumulative learning gain, the latter correcting for the participant’s starting level (performance on the first trial in the first session). In the figure pursuit, the cumulative learning gain was calculated as (T1 + T2 + T3 + T4 + T5 + T6 + T7 + T8)/8-S1T1. In circle pursuit, this was (T1 + T2)/2-S1T1.

We analyzed the PR data using a general linear model (GLM) with repeated measures. Because the time variable is accounted for by two separate variables in our study design, we first conducted an overall analysis with two within-subjects factors (SessionNumber, TrialNumber) and one between-subjects factor (Group, three levels). A *post hoc* analysis was used to contrast the three study groups, using Bonferroni correction to adjust for multiple comparisons (Analyses 1 and 2).

In the second step, we applied separate GLM repeated measures analyses to compare the learning over trials of groups Y–S and Y–E within the first session, which we expected to express the greatest learning effect. Subsequently, we compared the between-subjects effects of group in this analysis to the effects of group in a second analysis accounting for a covariate variable that was expected to influence the between-group differences (Analyses 3 and 4).

This procedure was repeated for three different covariates: LCT movement time (LCT_MT, an estimate of motor speed), WCST categories completed (WCST_cat, a measure of executive functioning), and education years.

In the third step, we used the computed learning measures (mean and cumulative learning gain) in separate GLM repeated measures analyses to evaluate the learning across sessions between groups Y–S and Y–E (Analyses 5 and 6).

## Results

### Demographics and baseline assessments

Demographic features, baseline assessment results and group differences are summarized in Table 2. Schizophrenia patients (S) had a significantly lower level of education and premorbid IQ compared to the young controls (Y), whereas the young controls and elderly participants (E) did not differ significantly for this parameter. The Y group significantly outperformed both E and S groups on the LCT and WCST measures (see Table 2). Composite symptom scores for schizophrenia patients were 25.67 ± 17.39 on the SANS scale and 14.24 ± 19.68 on the SAPS scale. A summary of the use of antipsychotic drugs in schizophrenia patients included in the study is provided in Table 3.

**Table 2:** **Demographic and baseline assessment results**.

	Schizophrenia patients (S)	Young controls (Y)	Elderly participants (E)	*T*-test S–Y	*T*-test E–Y
*N*	30	30	30	Matched	Matched
Age; mean (range)	36.4 (23–53)	37.3 (18–52)	69.2 (65–79)	*t*(58) = 0.16; *p* = 0.875	*t*(58) = 18.99; *p* < 0.001**
Sex M:F	20:10	20:10	20:10	Matched	Matched
Education years; mean (SD)	12.2 (±2.4)	15.1 (±2.6)	14.5 (±3.4)	*t*(58) = 4.50; *p* < 0.001**	*t*(58) = 0.74; *p* = 0.465
NLV Premorbid IQ; mean (SD)	101.30 (±10.29)	110.07 (±6.39)	111.73 (±6.43)	*t*(58) = 3.96; *p* < 0.001**	*t*(58) = 1.01; *p* = 0.318
LCT movement time; mean (SD)	0.36 (±0.15)	0.27 (±0.12)	0.40 (±0.13)	*t*(55) = 2.40; *p* = 0.020*	*t*(55) = 3.65; *p* = 0.001**
WCST categories completed; median (range)	3 (0–5)	5 (0–5)	3 (0–6)	*t*(58) = 2.60; *p* = 0.012*	*t*(56) = 3.35; *p* = 0.001**

*NLV, Dutch adult reading test/*Nederlandse Leestest voor Volwassenen*; LCT, line-copying task); WCST, Wisconsin card sorting test*.

*T*-test differences are reported as *t*(df) and *p*-values (*significant at the 0.05 level; **significant at the 0.01 level).

**Table 3:** **Antipsychotic drug prescriptions in schizophrenia patients**.

Antipsychotic drug name	Number of prescriptions	Dose range
Clozapine	8	50–700 mg/day
Amisulpiride	7	200–800 mg/day
Haloperidol decanoate	7	75–200 mg/month
Quetiapine	6	50–600 mg/day
Olanzapine	3	5–20 mg/day
Paliperidone	3	3–6 mg/day
Paliperidone depot	3	75–200 mg/month
Aripiprazole	2	10–30 mg/day
Olanzapine depot	2	210–405 mg/month
Risperidone depot	2	50 mg/month
Clotiapine	1	40 mg/day
Flupentixol	1	1 mg/day
Bromperidol decanoate	1	125 mg/month
Zuclopentixol depot	1	200 mg/month
Risperidone	1	4 mg/day

### Analyses of circle and figure pursuit

#### Learning the PR tasks over all trials and sessions

In Analysis 1, independent of groups, learning effects were demonstrated by significant main effects of TrialNumber and SessionNumber and a significant interaction TrialNumber* SessionNumber both in the CPR task and the FPR task, indicating that the learning curves over trials for each session were different [see Table 4A].

**Table 4:** **Results of the GLM repeated measures analyses**.

		Figure pursuit		Circle pursuit	
		*F* (hypothesis df, error df)^a^	*p*	*F* (hypothesis df, error df)^a^	*p*
**(A) Y, S, AND E GROUPS**					
SessionNumber^b^		285.76 (2, 88)	<0.001**	173.36 (2, 88)	<0.001**
TrialNumber^b^		179.77 (7, 83)	<0.001**	140.82 (1, 89)	<0.001**
SessionNumber*TrialNumber^b^		4.17 (14, 76)	<0.001**	13.34 (2, 88)	<0.001**
Group^c^		31.59	<0.001**	30.80	<0.001**
SessionNumber*TrialNumber*Group^c^		1.97 (28, 148)	0.005**	0.54 (4, 172)	0.710
**(B) SESSION 1, Y GROUP – S GROUP**					
TrialNumber*Group^d^		2.80 (7, 52)	0.015*	0.07 (1, 58)	0.799
Group^d^		7.80	0.007**	6.51	0.013*
**WITH COVARIATE**					
WCST_cat	Covariate^e^	19.42	<0.001**	9.35	0.003**
	Group^e^	4.57	0.114	2.54	0.117
					
LCT_MT	Covariate^e^	7.32	0.009**	3.15	0.082
	Group^e^	2.98	0.090	3.03	0.087
					
Education years	Covariate^e^	4.97	0.030*	2.18	0.146
	Group^e^	1.82	0.183	1.84	0.181
					
WCST(1) + LCT_MT(2)	Covariate1^e^	16.33	<0.001**		
	Covariate2^e^	5.84	0.010**		
	Group^e^	0.67	0.418		
					
Education years (1) + LCT_MT(2)	Covariate1^e^	5.48	0.023*		
	Covariate2^e^	8.86	0.004**		
	Group^e^	0.17	0.685		
**SESSION 1, Y GROUP – E GROUP**					
	TrialNumber*Group^d^	3.42 (7, 52)	0.004**	0.08 (1, 58)	0.775
	Group^d^	85.77	<0.001**	43.63	<0.001**
**WITH COVARIATE**					
WCST_cat	Covariate^e^	9.01	0.004**	6.84	0.011*
	Group^e^	61.35	<0.001**	27.38	<0.001**
LCT_MT	Covariate^e^	5.24	0.024*	0.61	0.439
	Group^e^	52.51	<0.001**	27.55	<0.001**
**(C) LEARNING MEASURE OVER SESSIONS**					
Mean	Y, S, and E groups				
	SessionNumber*Group^f^	2.51 (4, 172)	0.043*	1.59 (4, 172)	0.179
	Group^f^	31.59	<0.001**	30.80	<0.001**
	Y group – S group				
	SessionNumber*Group^f^	0.10 (2, 57)	0.905	0.87 (2, 57)	0.424
	Group^f^	9.16	0.004**	11.74	0.001**
	Y group – E group				
	SessionNumber*Group^f^	3.42 (2, 57)	0.039*	3.19 (2, 57)	0.049*
	Group^f^	83.78	<0.001**	70.26	<0.001**
Cumulative learning gain	Y, S, and E groups				
	SessionNumber*Group^g^	2.51 (4, 172)	0.043*	1.59 (4, 172)	0.179
	Group^g^	7.81	0.001**	1.54	0.220
	Y group – S group				
	SessionNumber*Group^g^	0.10 (2, 57)	0.905	0.87 (2, 57)	0.424
	Group^g^	1.04	0.312	1.50	0.225
	Y group – E group				
	SessionNumber*Group^g^	3.42 (2, 57)	0.039*	3.19 (2, 57)	0.049*
	Group^g^	16.23	<0.001**	0.269	0.107

*^a^Wilk’s Lambda F for multivariate analysis results*.

*^b^Analysis 1: within-subjects factors SessionNumber(3) and TrialNumber (8 FPR; 2 CPR)*.

*^c^Analysis 2: within-subjects factors SessionNumber(3) and TrialNumber (8 FPR; 2 CPR) and between-subjects factor Group (3)*.

*^d^Analysis 3: within-subjects factor TrialNumber (8 FPR; 2 CPR) and between-subjects factor Group (2)*.

*^e^Analysis 4: within-subjects factor TrialNumber (8 FPR; 2 CPR), between-subjects factor Group (2) and covariate*.

*^f^Analysis 5: within-subjects factor SessionNumber(3), between-subjects factor Group (3 or 2), variable mean*.

*^g^Analysis 6: within-subjects factor SessionNumber(3), between-subjects factor Group (3 or 2), variable cumulative learning gain*.

*Significant at the 0.05 level; **significant at the 0.01 level.

Upon addition of Group as between-subjects factor in analysis 2, in both PR tasks, a significant main effect of Group was found [see Table 4A]. *Post hoc* analysis with Bonferroni correction demonstrated that the performance of schizophrenia patients and elderly participants was significantly poorer than the young control subjects at all stages of the task (*p* < 0.001), and that the elderly participants were the worst performing group (E-S CPR mean difference −13.62, SE 3.11, *p* < 0.001; E-S FPR mean difference −14.42, SE 2.97, *p* < 0.001).

**Figure 3:**
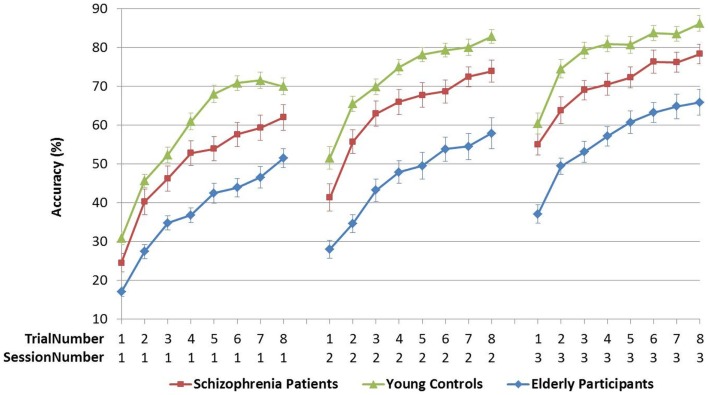
**Figure pursuit accuracy over three sessions and eight trials**.

**Figure 4:**
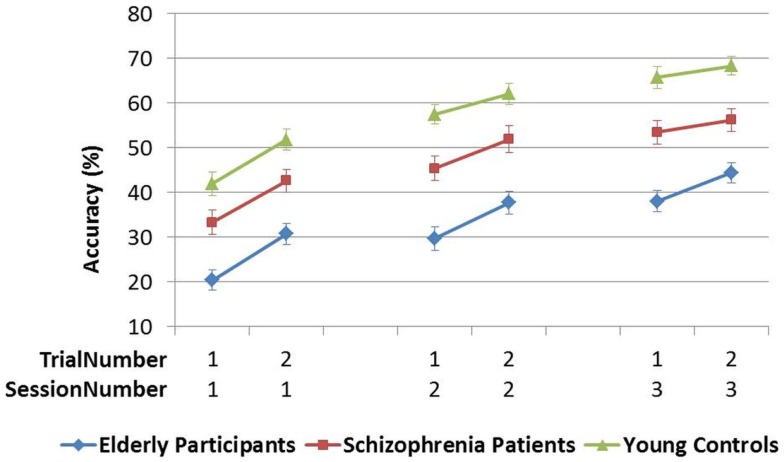
**Circle pursuit accuracy over three sessions and two trials**.

In contrast, the interaction of TrialNumber*SessionNumber* Group was only significant in FPR, indicating that the learning curves of the groups also followed different slopes (with significant linear, quadratic, and cubic components). In CPR, the slopes were similar for the three groups [see Table 4A; Figures 3 and 4].

#### Learning the PR tasks over trials within session 1

In Analysis 3, comparisons of groups Y–S and Y–E on the FPR session 1 showed both a difference in performance, indicated by the significant between-subjects effect of the Group variable, as well as a different learning slope over trials, indicated by the significant TrialNumber*Group interaction. However, the significant Y–S group effect was reduced to a non-significant value when accounting for significant covariates: LCT_MT, WCST_cat, and education years in Analysis 4 [see Table 4B]. Combination of two individually significant covariates (LCT_MT plus WCST_cat and LCT_MT plus education years) further reduced the FPR Group effect.

In CPR session 1, again there was only a significant between-subjects effect of Group without TrialNumber*Group interaction. Furthermore, only the WCST_cat covariate reached a level of significance in the between-groups effect in this task, reducing also the Group difference between Y and S to a non-significant level [see Table 4B].

Interestingly, when these same covariates were added to the Y–E comparison, in both PR tasks the between-subjects Group effect remained significant [see Table 4B].

None of the analyses with covariates demonstrated a significant TrialNumber*Covariate or Group*Covariate interaction, suggesting the main effects of the covariates on the Accuracy variable can be interpreted independently of Group or Trialnumber.

#### Learning the PR tasks over sessions

**Figure 5:**
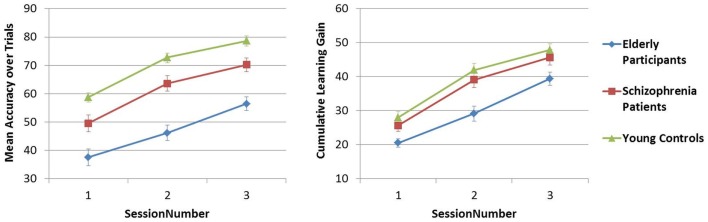
**Figure pursuit mean accuracy and cumulative learning gain over three sessions**.

**Figure 6:**
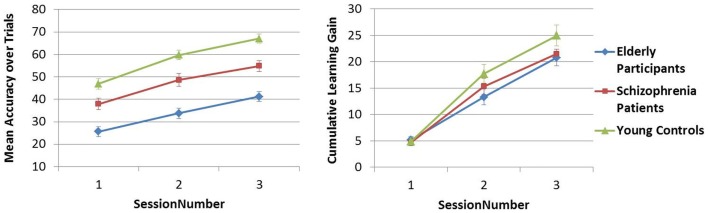
**Circle pursuit mean accuracy and cumulative learning gain over three sessions**.

The mean accuracy over trials was compared across sessions 1–3 in Analysis 5. In both PR tasks, a difference in performance between Y–S and Y–E groups was observed (i.e., significant main between-subjects effect of Group), but the SessionNumber*Group interaction was only significant for Y–E comparison, suggesting that schizophrenia patients showed a similar learning pattern across sessions as did young controls, but elderly participants did not [see Table 4C; Figures 5 and 6].

In Analysis 6, the same analyses were repeated with the learning measure cumulative learning gain, which corrects the mean for the participant’s starting level performance on the first trial in session 1. Here, when Y and S groups were compared, neither the SessionNumber*Group interaction nor the between-subjects effect of Group was significant in either of the PR tasks. In the comparison of Y and E groups, a significant interaction of SessionNumber*Group was maintained for both PR tasks, but the main effect of Group was only significant for FPR [see Table 4C; Figures 3 and 4].

## Discussion

### Key results

#### General performance

Our results demonstrate poorer performance both in schizophrenia patients and in elderly participants compared to young controls, thereby matching findings of previous rotary pursuit studies (8, 11, 12, 14, 15, 17). This finding was observed in both pursuit tasks, and both on a within- and across-session level.

In FPR session 1, the poorer performance in patients was found to be attributable to differences in other functional parameters, such as psychomotor speed (LCT_MT), executive functioning (WCST categories completed), and years of education, with an additive effect. This implies that patients performing worse than healthy controls on the FPR task also perform worse on one or several of these baseline measures. One could thus hypothesize that the impaired FPR performance of patients is caused by reduced psychomotor speed and/or executive functioning or a lower level of education. Alternatively, it could also point out the existence of a separate subgroup of schizophrenia patients exhibiting impairments on all of these domains. In contrast, the difference in FPR performance between elderly participants and young controls could not be accounted for by differences in psychomotor speed nor executive functioning, indicating a performance gap between these groups that is independent of other functional parameters.

In CPR, contrasting to what was expected, psychomotor speed did not have a significant effect on the performance in session 1. Only executive functioning level appeared to account significantly for the difference between schizophrenia patients and controls in this task.

After the mean performance per session was corrected for the initial starting level, there was no longer a significant difference in performance across sessions between patients and controls. This finding suggests that the lower mean performance of patients is caused by a significantly lower starting level, which is not recovered by additional practice. However, in two other recent PR studies, an individual equation of the target speed was applied to account for participants’ starting level performance; yet, the general performance in schizophrenia patients was found to be impaired nonetheless (12, 15). Thus, adjusting the difficulty of the task does not seem to solve the performance gap. Further study is needed to understand these seemingly contradictory findings. Regarding the elderly participants, their poorer mean level of performance was amended in the CPR, but remained in the FPR after correction for their significantly lower starting level by the cumulative learning gain measure.

#### Skill learning rate

We have established that all groups learned the new FPR and CPR sensorimotor skills over trials and sessions, but whereas the overall learning rate of schizophrenia patients and elderly participants was preserved in CPR, it differed between the three groups in FPR.

The early phase of learning the FPR skill was characterized by a significantly different learning curve of the schizophrenia patients and the elderly participants compared to young controls, who reached their peak performance earlier, as illustrated in Figure 3. The CPR consisted of only two trials in the first session, and therefore by definition the learning rate was marked by a linear increase, of which the slopes did not differ between the three groups (see Figure 4). In the later phase of learning of both PR tasks, schizophrenia patients and control subjects showed comparable learning gains over sessions, but elderly participants learned significantly less.

### Study limitations

By using two variations of the PR task, we have attempted to distinguish motor and sequence learning components, yet it remains difficult to single out and evaluate separately the processes involved in sensorimotor learning and performance. We cannot rule out the impact of declarative and spatial memory, attention capacity, and motor coordination on our PR skill performance and learning results. Also, while our CPR task was similar to the classical rotary pursuit task, we used a different methodology regarding the number of trials and rotation speed (see Table 1), which complicates the comparison of our results in the CPR to those of previous PR studies. It is possible that the number of trials per session in our CPR was too limited to establish within-session learning differences, which were found in FPR but not in CPR.

This study included only schizophrenia outpatients who were able to complete the test batteries and results can, therefore, not necessarily be generalized to the whole population of patients with schizophrenia. However, the mean SAPS and SANS composite scores in our sample concurred with scores found by van Erp et al. in a sample of 205 schizophrenia patients: mean composite SAPS 16.8 ± 14.2 compared to 14.2 ± 19.7 in our sample, and mean composite SANS 23.0 ± 14.6 compared to 25.7 ± 17.4 in our sample (21). Our patient population can, therefore, not be presumed to differ significantly in terms of symptom severity from other schizophrenia patient samples.

A large within-group heterogeneity in performance existed, particularly in the starting performance level of schizophrenia patients and the final performance level of elderly subjects. The relatively higher performance heterogeneity of the patients and elderly participants compared to the young controls may imply performance on the PR tasks in these groups was influenced by other variables than those accounted for in our study design. Problems with the evaluation of cognition of schizophrenia patients include lack of motivation and attention problems caused by negative symptomatology. Patients were instructed to complete the tasks to the best of their ability and our experience during test procedures was that being able to follow the feedback of their performance live on-screen provided an additional stimulus for performance optimization to subjects in all groups.

Other variables that may affect task performance include general cognitive functioning and medication use. We did not evaluate the study groups for their current IQ scores, and the schizophrenia patients had a significantly lower premorbid IQ score compared to young and elderly controls. In previous studies, comparing IQ-matched subgroups reduced or abolished differences in the overall level of performance between schizophrenic patients and control subjects on the rotor pursuit and other tasks of procedural learning (14, 15). However, IQ matching may also introduce a bias, considering research of general intelligence in schizophrenia has shown that only about a quarter of schizophrenia patients have a preserved IQ compared to the general population (22). Moreover, correlations between motor and cognitive functioning in schizophrenia patients have been repeatedly demonstrated (23, 24), and matching for cognitive parameters in studies of motor learning may, therefore, greatly influence the primary outcome measure. It is often argued that cognitive impairments in schizophrenia, and specifically psychomotor ones, are caused by psychotropic substances in general and antipsychotic medication in particular. All patients in our study had been stably treated with antipsychotic medication at the time of testing, and 16 patients were using more than one antipsychotic drug concomitantly (see Table 3). The reduced performance on PR tasks combined with a normal learning rate in patients may be hypothesized to be due to the use of antipsychotic drugs, known to affect psychomotor functioning (25).

### Implications for future research and clinical perspectives

Our study provides important caveats toward future research on procedural learning in schizophrenia. Researchers should be aware that motor tasks including a sequence component should be distinguished from true motor learning tasks. As shown in this study, small variations applied to commonly used procedural tasks may allow to distinguish between operationally different components that may be important to further elucidate the nature of the deficits in schizophrenia. Particularly, the combination of different sensorimotor learning tasks with imaging techniques can be valuable to evaluate structural and functional brain alterations in the motor system. Furthermore, a longitudinal design should be a key feature of any study design interested in aspects of learning and memory, with differentiation of early and late learning phases.

Cognitive functioning, and specifically also executive functioning as measured with the Wisconsin card sorting test, has been shown to be a major predictor of functional outcome in schizophrenia (1). Motor learning in schizophrenia has been less studied, and its relation to functional outcome is currently unknown. However, evidence that motor performance is not only related to cognitive and executive functioning but also a predictor of cognitive deficits in schizophrenia patients at 1-year follow-up (23) suggests an association between motor performance or learning capacity, and functional outcome may exist that merits further investigation.

An age-related decline in sensorimotor learning has been previously recognized (17, 26), and in our study indeed the elderly participants demonstrated both poorer performance and lower learning gains in both PR tasks. Unexpectedly, the schizophrenia patients even outperformed the elderly healthy participants. Although this finding needs to be confirmed, it governs a more optimistic message about the functioning of patients than has hitherto been assumed. However, it is uncertain whether this pattern is maintained throughout different cognitive domains. Findings of our research group, as reported elsewhere in this journal (Cornelis et al., in press), suggest that in other cognitive domains, elderly participants may outperform schizophrenia patients. It might be interesting for future studies to include both elderly and non-elderly schizophrenia and control participants to differentiate between the mechanisms of cognitive impairment related to aging and schizophrenia.

A generally lower level of performance in schizophrenia (starting and ending the learning phase at a lower level than control subjects) has been a frequent finding in PR studies (11, 12, 14). Some authors have interpreted this phenomenon as reflecting impaired procedural learning in schizophrenia patients. However, since this reduced overall level of performance is usually accompanied with a normal learning rate, the mechanisms that underlie these two aspects of task performance are likely to differ to some extent. Because of this difficulty to differentiate between y-intercept (absolute performance) and slope (learning rate), and because of the high degree of within-group heterogeneity on performance level, many studies have not been able to conclude as to the actual capacity for sensorimotor skill learning of schizophrenia patients. Based on our results, it seems that schizophrenia patients have a mostly preserved capacity to learn sensorimotor skills, with any deficit related more to the early learning phase of sequence-holding skills and depending largely on the starting level performance of patients. This knowledge may prove important to the development and evaluation of therapies to improve such deficits in schizophrenia, in which the rotary pursuit, a well-established, easy and quick to administer task, may be used for the initial and follow-up evaluation of motor learning capacity and performance in patients. Because the late-phase learning of patients was preserved, it can be suspected that with an extended number of trials, the patients could eventually reach the same performance level as the final level in young controls. Thus, schizophrenia patients maintain the ability to acquire new skills, of vital importance to everyday functioning, given extra room for rehearsal. On the other hand, since more complex skills often also require additional cognitive components related to planning and organization, it is unclear whether this finding may be translated to all real-life skills.

## Conclusion

Both in circle pursuit (motor task) and figure pursuit (motor plus sequence task), learning was evident in all groups, with equal learning gains of schizophrenia patients compared to age-matched controls, but reduced learning in elderly participants. In terms of general performance, the schizophrenia patients fell between the young controls and the elderly participants, differing significantly from both. Our results suggest that the lower performance of schizophrenia patients compared to age-matched controls can be accounted for by impaired speed of movement and executive functioning.

## Author Contributions

All the authors met ICMJE criteria and all those who fulfilled those criteria were listed as authors. All the authors had access to the study data and made the final decision about where to present these data.

## Conflict of Interest Statement

This clinical trial received funding support by Janssen Research & Development, a division of Janssen Pharmaceutica N.V., Beerse, Belgium. The co-authors Luc Janssens and Maarten Timmers are employees of Janssen Research & Development, a division of Janssen Pharmaceutica N.V., Beerse, Belgium, and own stock/stock options in the company.

## Abbreviations

CPR, circle pursuit rotor; E group, elderly participants; FPR, figure pursuit rotor; GLM, general linear model; LCT, line-copying task; MT, movement time; NLV, *Nederlandse Leestest voor Volwassenen;* PR, pursuit rotor/rotary pursuit; RPM, rotations per minute; S group, schizophrenia patients; WCST, Wisconsin card sorting test; Y group, young controls.
